# Redox Signaling Regulated by Cysteine Persulfide and Protein Polysulfidation

**DOI:** 10.3390/molecules21121721

**Published:** 2016-12-15

**Authors:** Shingo Kasamatsu, Akira Nishimura, Masanobu Morita, Tetsuro Matsunaga, Hisyam Abdul Hamid, Takaaki Akaike

**Affiliations:** Department of Environmental Health Science and Molecular Toxicology, Tohoku University Graduate School of Medicine, Sendai 980-8575, Japan; shingok@med.tohoku.ac.jp (S.K.); Akira.Nishimura@med.tohoku.ac.jp (A.N.); morita@med.tohoku.ac.jp (M.M.); matsunag@med.tohoku.ac.jp (T.M.); hisyamabdulhamid@gmail.com (H.A.H.)

**Keywords:** reactive persulfide species, protein polysulfidation, thiol modification, redox signaling

## Abstract

For decades, reactive persulfide species including cysteine persulfide (CysSSH) have been known to exist endogenously in organisms. However, the physiological significance of endogenous persulfides remains poorly understood. That cystathionine β-synthase and cystathionine γ-lyase produced CysSSH from cystine was recently demonstrated. An endogenous sulfur transfer system involving CysSSH evidently generates glutathione persulfide (GSSH) that exists at concentrations greater than 100 μM in vivo. Because reactive persulfide species such as CysSSH and GSSH have higher nucleophilicity than parental cysteine (Cys) and glutathione do, these reactive species exhibit strong scavenging activities against oxidants, e.g., hydrogen peroxide, and electrophiles, which contributes to redox signaling regulation. Also, several papers indicated that various proteins and enzymes have Cys polysulfides including CysSSH at their specific Cys residues, which is called protein polysulfidation. Apart from the redox signaling regulatory mechanism, another plausible function of protein polysulfidation is providing protection for protein thiol residues against irreversible chemical modification caused by oxidants and electrophiles. Elucidation of the redox signaling regulatory mechanism of reactive persulfide species including small thiol molecules and thiol-containing proteins should lead to the development of new therapeutic strategies and drug discoveries for oxidative and electrophilic stress-related diseases.

## 1. Introduction

**Figure 1:**
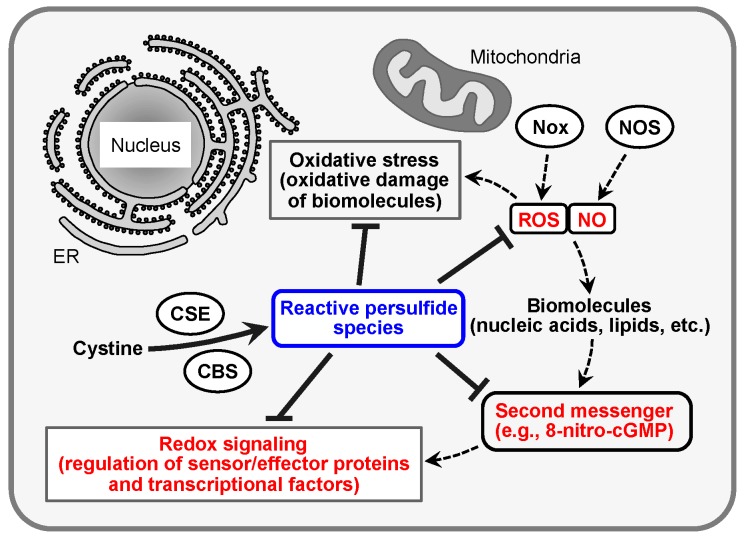
Regulation of oxidative stress and redox signaling by reactive persulfide species. Excess production of ROS and NO from Nox and NOS during infection and inflammation causes oxidative stress. In contrast, ROS and NO also function as redox signals via formation of electrophilic second messengers such as 8-nitro-cGMP. Reactive persulfide species, synthesized by CBS and CSE, regulate oxidative stress and redox signal pathways by the metabolism of ROS and NO and the electrophilic second messenger. ROS, reactive oxygen species; NO, nitric oxide; Nox, NADPH oxidase; NOS, NO synthase; 8-nitro-cGMP, 8-nitroguanosine 3′,5′-cyclic monophosphate; CBS, cystathionine β-synthase; CSE, cystathionine γ-lyase; ER, endoplasmic reticulum.

Reactive oxygen species (ROS) such as superoxide anion (O_2_^•−^) and hydrogen peroxide (H_2_O_2_) form after incomplete reduction of molecular oxygen during the defense response to infection and inflammation or as by-products of mitochondrial respiration in cells. That ROS as potent oxidants can mediate the toxicity of oxygen (oxidant toxicity theory) is conceivable because of their greater chemical reactivity compared with that of molecular oxygen. Indeed, these reactive molecular species, in concert with nitric oxide (NO), which is produced by NO synthase (NOS), participate in different diseases caused by oxidative stress [1,2,3,4,5]. Despite their potential pathogenicity, however, they can function as intracellular signaling molecules via reaction with various biological molecules including nucleic acids and lipids to form more stable electrophilic molecules (e.g., 8-nitroganosine 3′,5′-cyclic monophosphate, 8-nitro-cGMP) (Figure 1) [4,6,7,8,9,10,11,12]. Further, 8-nitro-cGMP reacts with protein thiol residues to add the cGMP structure to the protein through a process called *S*-guanylation. *S*-Guanylation of sensor and effector proteins is associated with several important biological processes [6,9]. However, the regulatory mechanism of this unique electrophile-mediated redox signaling is not yet fully understood.

We recently found endogenous production of the reactive persulfide species such as cysteine persulfide (CysSSH) and glutathione persulfide (GSSH) in cells and tissues and demonstrated that these molecules play a critical role in the regulation of 8-nitro-cGMP signaling [13,14]. Additional sulfur atoms in reactive persulfide species enhance their nucleophilicity and antioxidant ability compared with the commonly known thiol molecules [13,14]. Cysteine (Cys) thiol residues of many proteins were also demonstrated to contain a polysulfide structure (-S-(S)_n_-H) (i.e., protein polysulfidation), which led us to expect that reactive persulfide species play important roles in the regulation of redox signaling [13,14,15,16,17,18,19,20]. In this review article, we provide a brief overview of the redox signaling mechanism of ROS and the second messenger 8-nitro-cGMP, and we create a new paradigm adapted from conventional oxidant toxicity theory. Moreover, we elaborate on the mechanisms of production and functions of reactive persulfide species including low-molecular-weight (LMW) compounds and protein polysulfides from the point of view of redox signal regulation.

## 2. Redox Signaling and Its Second Messenger 8-Nitro-cGMP

An excess of ROS and NO is generated in cells during infection and inflammation. For example, in cells and tissues exposed to inflammatory conditions, NADPH oxidase (Nox) [21,22] and the mitochondrial electron transport chain produce ROS such as O_2_^•−^ and H_2_O_2_, whereas NO is produced by inducible NOS from molecular oxygen and l-arginine. ROS react spontaneously with NO to form reactive nitrogen oxide species (RNOS) such as peroxynitrite (ONOO^−^), which have much greater chemical reactivity compared with ROS and NO [10]. Because of their high reactivity, ROS and RNOS can react and trigger chemical modifications such as oxidation, nitration, and nitrosation in various biological molecules including proteins, nucleic acids, and lipids (Figure 1). These nonspecific modifications are thought to be oxidative damage of biological molecules (oxidative stress) and result in impaired cellular processes. However, one concept proposed that relatively stable ROS such as H_2_O_2_ may be physiologically synthesized as normal aerobic metabolites and have physiological functions in cells [23,24,25,26]. In view of this proposal, our understanding of the concept that ROS mainly cause nonspecific injury of biomolecules shifted to a broader perspective. In fact, various researchers reported that primary ROS and downstream electrophiles are produced by the chemical modification of biological molecules by ROS and RNOS and function in physiological signaling (redox signaling) in cell responses related to oxidative stress (Figure 1) [4,6,7,8,9,10,11,12]. Further, 8-nitro-cGMP was identified some years ago and was reported in 2007 to be a second messenger in ROS and NO signaling during oxidative stress resulting from infection and inflammation [6]. This electrophilic nucleotide is produced by soluble guanylate cyclase in mammalian cells and adenylate cyclase in bacterial cells from the nitrated nucleotide 8-nitroguanosine triphosphate, which is generated by reaction with ONOO^−^ and GTP, as an enzymatic substrate [9,27,28]. In addition, 8-nitro-cGMP can activate cGMP-dependent protein kinase (PKG) and induce vasorelaxation similarly to the common NO second messenger cGMP [6]. In fact, 8-nitro-cGMP has a longer half-life under physiological conditions than the corresponding cGMP because of its resistance to degradation by phosphodiesterases (PDEs) [6].

**Figure 2:**
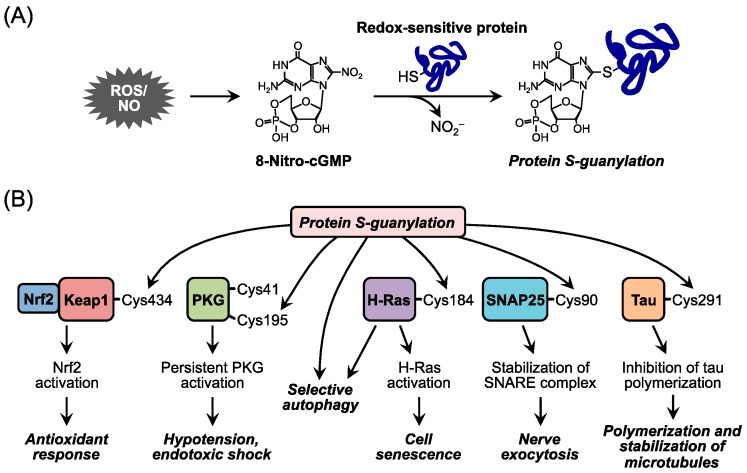
Redox signal transduction of 8-nitro-cGMP via protein *S*-guanylation. (**A**) Protein *S*-guanylation by 8-nitro-cGMP. The 8-nitro-cGMP, a nitrated cyclic nucleotide, is electrophilic and causes *S*-guanylation of proteins via reaction with redox-sensitive thiols on proteins; (**B**) Biological significance of protein *S*-guanylation. *S*-Guanylation of individual proteins is involved in various cellular processes. 8-Nitro-cGMP, 8-nitroguanosine 3′,5′-cyclic monophosphate; NO_2_^−^, nitrite; Keap1, Kelch-like ECH-associated protein 1; PKG, cGMP-dependent protein kinase; SNAP25, synaptosomal-associated protein 25.

Moreover, this nitrated cyclic nucleotide exhibits a unique posttranslational protein modification: addition of the cGMP structure to a specific Cys residue in proteins via its electrophilicity derived from the nitro group. This modification is called *S*-guanylation (Figure 2). Our research group and others identified several target proteins of *S*-guanylation and reported their biological significance [6,8,9,27,28,29,30,31,32,33,34]. For example, *S*-guanylation of Keap1 (Kelch-like ECH-associated protein 1), an oxidative stress sensor protein, activates transcriptional factor Nrf2 and induces the expression of antioxidant enzymes such as heme oxygenase 1, which in turn exerts cytoprotective effects [6,8,9]. Also, septic shock–induced 8-nitro-cGMP reacts with PKG and continuously *S*-guanylates PKG, which results in persistent vasodilation and hypotensive shock [32]. The small G protein H-Ras, in contrast, is simultaneously activated by *S*-guanylation and induces senescence of cardiac cells, which leads to chronic heart failure [29]. *S*-Guanylation of proteins in group A streptococcus has been verified as functioning as a biomarker for selective autophagic degradation of the bacteria and immunoreaction in the host cells [30]. Certain studies reported that *S*-guanylation of synaptosomal-associated protein 25 and tau protein induced by endogenous 8-nitro-cGMP may play a role in the regulation of neuronal exocytosis and the pathogenic mechanisms of neurodegenerative diseases, for example Alzheimer’s disease [31,33]. These various physiological functions of 8-nitro-cGMP highlight its diversity in modulating the redox signaling of ROS and RNOS. Elucidation of the underlying mechanism of action of 8-nitro-cGMP is expected to reveal more functions not only involved in cellular stress responses during infection and inflammation and other disease conditions but also related to other fundamental physiological cellular events.

## 3. CysSSH and Other Reactive Persulfide Species

**Figure 3:**
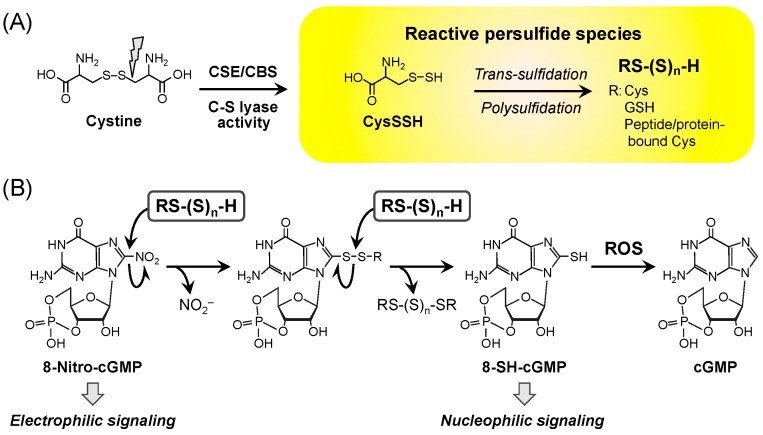
Endogenous formation of reactive persulfide species and its function in 8-nitro-cGMP metabolism. (**A**) Formation of reactive persulfide species by CBS and CSE. CBS and CSE produce CysSSH as a primary product from cystine, and the following reaction with endogenous thiol compounds forms various reactive persulfide species in vivo; (**B**) Reactive persulfide species–dependent metabolic pathway regulating 8-nitro-cGMP signaling. The 8-nitro-cGMP reacts with reactive persulfide species to form 8-SH-cGMP with the release of nitrite. An additional reaction between the sulfhydrated metabolite 8-SH-cGMP and ROS results in cGMP formation by oxidative desulfidation, and the cGMP thus formed is then degraded by phosphodiesterase. CBS, cystathionine β-synthase; CSE, cystathionine γ-lyase; Cys, cysteine; CysSSH, cysteine persulfide; GSH, glutathione; cGMP, guanosine 3′,5′-cyclic monophosphate; 8-nitro-cGMP, 8-nitroguanosine 3′,5′-cyclic monophosphate; NO_2_^−^, nitrite anion; ROS, reactive oxygen species.

During our analysis of the details of 8-nitro-cGMP metabolism in vivo, our group identified endogenous and abundant formation of the reactive persulfide species CysSSH and GSSH in biological systems. By means of RNA interference screening, we found that cystathionine β-synthase (CBS) and cystathionine γ-lyase (CSE) are involved in critical ways in 8-nitro-GMP metabolism [13,29]. Although these two enzymes are implicated in the production of hydrogen sulfide (H_2_S), extensive biochemical analyses with liquid chromatography-tandem mass spectrometry (LC-MS/MS) showed that H_2_S itself apparently cannot metabolize 8-nitro-cGMP. Nonetheless, these enzymes demonstrated enzymatic activity and effectively produced CysSSH from cystine (Figure 3) [13]. Cavallini et al. first reported that CSE utilizes cystine as a substrate for its C-S lyase activity, and the subsequent persulfide-related CSE studies suggested that CSE may generate persulfides that may contribute to the regulation of redox-related metabolism [35,36,37,38,39]. Notably, the CysSSH formed is highly nucleophilic and a stronger antioxidant compared with Cys [13]. These unique chemical properties of CysSSH rely on adjacent electron pairs, an effect that is known as the α-effect [14,40]. Because of its high nucleophilicity, CysSSH behaves as a typical reactive persulfide species and reacts quite efficiently with 8-nitro-cGMP to form its sulfhydrated metabolite 8-SH-cGMP, with the release of nitrite (Figure 3B), which suggests that the reactive persulfide species regulate electrophilic signal transduction via the nucleophilic substitution of the electrophilic 8-nitro moiety of 8-nitro-cGMP [13]. As Figure 3 illustrates, two unique second messengers, i.e., 8-nitro-cGMP and 8-SH-cGMP, which are endogenously derived from ROS and NO and are modulated by reactive persulfide species such as CysSSH, may mediate signal transduction in a manner that reflects the opposite of their redox properties, electrophilic- and nucleophilic-based signaling, respectively. In fact, the endogenous formation of 8-SH-cGMP has been well documented in cultured mammalian cells and mouse tissues as well as in plant cells [13,41]. Also, 8-SH-cGMP shows resistance to PDE while retaining cGMP activity for PKG activation. The 8-SH-cGMP possesses a mercapto moiety that is removable in reactions with biologically relevant oxidants such as H_2_O_2_ and ONOO^−^, which results in the formation of cGMP as a primary product (Figure 3B) [29,42]. In biological systems, cGMP thus formed may be degraded by the action of PDEs. Therefore, sulfidation of 8-nitro-cGMP followed by oxidant-dependent desulfidation may contribute to the physiological decomposition of 8-nitro-cGMP and thus terminate electrophile signaling, as discussed above.

Reactive persulfide species can react with environmental electrophiles such as methylmercury (MeHg) and the endogenous electrophile 8-nitro-cGMP. As was demonstrated earlier, MeHg interacts with reactive persulfide species including GSSH, glutathione (GSH) polysulfide, and the authentic polysulfide Na_2_S_4_ to form bismethylmercury sulfide (MeHg)_2_S, which is a detoxified metabolite of MeHg [43,44]. In addition, we found that sulfide adducts of *N*-acetyl-*p*-benzoquinone imine, an electrophilic metabolite of acetaminophen, are produced in acetaminophen-exposed mice [45]. Thus, we propose a new concept: reactive persulfide species provide cytoprotective value against endogenous and exogenous electrophilic insults, and excessive exposure to electrophiles depletes these reactive species, which leads to critical vulnerability to electrophile-dependent toxicity.

Trans-sulfidation of CysSSH to GSH provides a pool of reactive persulfide species in cells and tissues that are present mainly as persulfides and polysulfides of GSH at various levels (Figure 3) [13,14]. As mentioned earlier, these reactive persulfide species may be responsible for regulation of oxidative stress as a part of the antioxidant response in vivo. In fact, in vitro experiments showed that GSH has little effect on the elimination of H_2_O_2_, whereas GSSH scavenges H_2_O_2_ quite effectively [13]. In addition, LC-MS/MS analysis to detect intercellular reactive persulfide species revealed that H_2_O_2_ treatment decreased more GSSH than GSH in amounts in cultured cells [13]. Also, the overexpression of CSE, a primary enzyme that produces reactive persulfide species, protects against H_2_O_2_-induced cell death [13]. Peroxidase enzymes are well known to scavenge H_2_O_2_ efficiently. Since it is known that reactive persulfide species can react with heme proteins [14], we consider that the reactive species may contribute to potent antioxidant effects in concert with peroxidases by promoting a heme-catalyzed peroxidation reaction via a persulfide-dependent pathway. In view of all the factors mentioned above, reactive persulfide species may have strong antioxidant effects in vivo and function cytoprotectively as a part of a defense mechanism against oxidative stress.

## 4. Protein Polysulfidation

**Figure 4:**
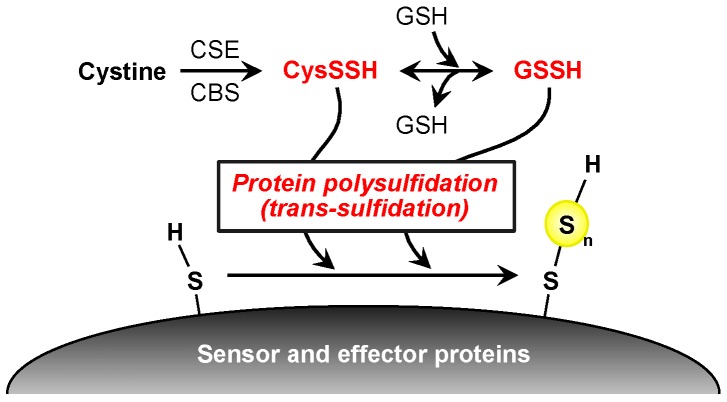
Possible mechanism of protein polysulfidation. CysSSH, generated by CBS and CSE, interacts with GSH to form GSSH, followed by a reaction with a thiol group on the redox sensor and effector proteins that forms protein polysulfide at a redox-sensitive residue. CysSSH, cysteine persulfide; CBS, cystathionine β-synthase; CSE, cystathionine γ-lyase; GSH, glutathione; GSSH, glutathione persulfide.

**Figure 5:**
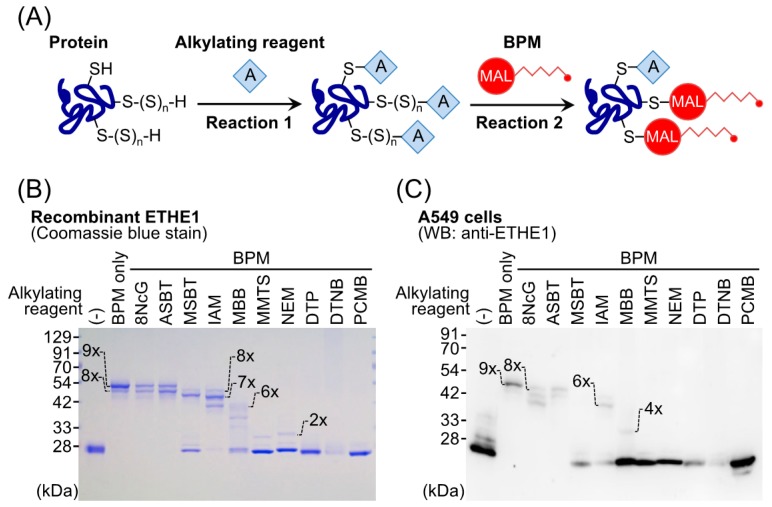
A new method to detect protein polysulfidation: the polyethylene glycol-conjugated maleimide-labeling gel shift assay (PMSA). (**A**) The principle of PMSA. In the initial reaction, an alkylating reagent blocks all thiol groups. In the second reaction, BPM labels polysulfidated Cys residues. The degree of protein polysulfidation is determined as a change in sodium dodecyl sulfate-polyacrylamide gel electrophoresis band mobility. Protein polysulfidation of recombinant ETHE1 (**B**) and endogenous ETHE1 in A549 cells (**C**). Numbers on the gels indicate the number of BPM labels in the protein, and thus the number of polysulfidated Cys residues. BPM, biotin-PEG-maleimide; MAL, maleimide; 8NcG, 8-nitroguanosine 3′,5′-cyclic monophosphate; ASBT, 2-aminosulfonyl benzothiazole; MSBT, 2-methylsulfonyl benzothiazole; IAM, iodoacetamide; MBB, monobromobimane; MMTS, methyl methanethiosulfonate; NEM, *N*-ethylmaleimide; DTP, 4,4′-dithiopyridine; DTNB: 5′-dithiobis(2-nitrobenzoic acid); PCMB, *p*-chloromercuribenzoic acid; Cys, cysteine; ETHE1, ethylmalonic encephalopathy protein 1; WB, Western blotting.

Recent studies reported that reactive persulfide species are present not only as LMW species but also as high-molecular-weight (HMW) species; that is, various enzymes and proteins have polysulfide moieties (called protein polysulfidation) at many Cys residues [13,18,19,20]. Transfer of a sulfur from LMW polysulfides such as GSSH to HMW protein-bound Cys residues may be a mechanism of protein polysulfidation (Figure 4) [13,14]. Indeed, we found that overexpression of CBS/CSE in the cells elevated intracellular GSSH levels as well as protein polysulfidation, which suggests trans-sulfidation mediated by these enzymes in mammalian cells. However, the detailed mechanism and the metabolic process of this reaction remain unclear. To study biological functions of protein polysulfidation, several research groups in the past decade developed different experimental procedures including modified tag-switch assays, MS-based methods, and a pull-down procedure [13,15,18,19,20]. These methods are based on the electrophilicity of polysulfide moieties of proteins, but our preliminary experiments showed that polysulfides possess a rather complex redox property, i.e., individual sulfide residues of various polysulfides are both nucleophilic and electrophilic. Therefore, we recently developed a convenient and selective method for detecting polysulfidated proteins by use of such a unique redox property of polysulfide, in which we used the probe biotin-polyethylene glycol-conjugated maleimide (biotin-PEG-MAL, BPM) and several alkylating reagents, the method being named the PEG-MAL-labeling gel shift assay (PMSA, Figure 5A) [46]. This technology enabled us to quantify protein polysulfide levels based on the difference in band mobility in sodium dodecyl sulfate-polyacrylamide gel electrophoresis. For example, as Figure 5B shows, we succeeded in detecting protein polysulfidation of ethylmalonic encephalopathy protein 1 (ETHE1, also called persulfide dioxygenase) by using the recombinant ETHE1 protein prepared with an *Escherichia coli* cell expression system. After BPM-only treatment (Figure 5B, lane 2), a band at a higher position (band fully shifted up) can be clearly seen. This result, however, was moderately affected by blocking with mild electrophilic reagents such as 8-nitro-cGMP (lane 3), 2-aminosulfonyl benzothiazole (lane 4), and iodoacetamide (lane 6) with almost the same or a slightly lower level of mobility. Groups pretreated with strong electrophilic reagents such as monobromobimane (lane 7), methyl methanethiosulfonate (lane 8), *N*-ethylmaleimide (lane 9), 4,4′-dithiopyridine (lane 10), 5′-dithiobis(2-nitrobenzoic acid) (lane 11), and *p*-chloromercuribenzoic acid (lane 12), however, showed bands at the lowest position, which suggests that these harsh electrophiles caused decomposition at the basal level of polysulfide structure and thus led to complete inhibition of the second reaction, BPM labeling. This PMSA demonstrated that the endogenous ETHE1 protein in A549 cells was also extensively polysulfidated at almost all Cys residues (Figure 5C). These findings clearly indicated that protein polysulfidation commonly occurs in *E. coli* and humans. Although our new method is quite convenient and simple, false-negative reactions may occur. The bands that were shifted up by BPM and mild electrophilic reagents may result from an incomplete blocking reaction. Therefore, confirmation with MS is required to unequivocally identify protein polysulfidation.

In the research field of redox biology, protein modifications at Cys residues are important for the regulation of protein functions [10]. A possible function of protein polysulfidation is protection of a sensitive Cys residue from irreversible oxidative or electrophilic modification [13,14]. In fact, although the covalent interaction of MeHg with protein thiols interrupts protein functions, protein polysulfides may avoid this modification by formation of (MeHg)_2_S as a product of the reaction between a protein polysulfide and MeHg [44]. In addition, Fukuto’s group recently reported that protein persulfidation may nullify oxidative and electrophilic modification of a Cys residue and lead to the recovery of the enzymatic activity of CD148 phosphatase [47].

**Figure 6:**
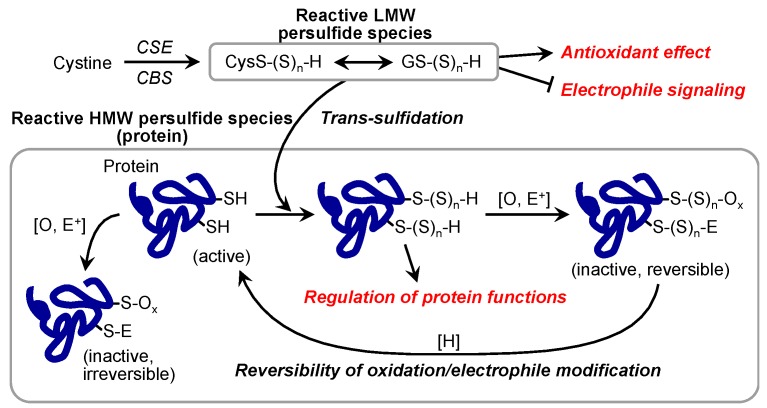
Possible functions of reactive persulfide species and protein polysulfidation. HMW, high molecular weight; LMW, low molecular weight; CBS, cystathionine β-synthase; CSE, cystathionine γ-lyase.

Our group and others reported protein polysulfidation of various proteins and enzymes and proposed some biological functions of such protein modifications (Figure 6) [15,46,48]. For example, ETHE1 may obtain additional persulfide residues from the substrate GSH polysulfide during its enzymatic reaction, thereby catalyzing the conversion of the GSH polysulfide to GSH and eliciting an extensive protein polysulfidation maintenance system [46]. Jarosz et al. reported that polysulfide treatment increased the polysulfidation of GAPDH protein at the Cys156 and Cys247 residues and that the modification inactivated the enzymatic activity [48]. Nuclear transcription factor Y subunit beta (NFYB) polysulfidation at Cys105 has been reported as important for the NFY complex binding to the promoters ten-eleven translocation methylcytosine dioxygenase 1 and 2 (Tet1 and Tet2, respectively) to regulate their expression [18]. Vandiver et al. reported that parkin, which is a component of a multiprotein E3 ubiquitin ligase complex and is closely related to Parkinson’s disease (PD), was physiologically polysulfidated in human and mouse brain and rat striatum and that this modification increased parkin activity [17]. As an interesting finding, polysulfidation of the parkin protein in the brain of patients with PD was markedly reduced, which suggested that this reduction may have pathological significance [17]. Indeed, treatment with polysulfide donors including H_2_S donors has had protective effects against PD [17,49]. Polysulfidation of Cu/Zn superoxide dismutase inhibited its oxidative-induced aggregation, which has been linked to some familial cases of amyotrophic lateral sclerosis [16,50,51]. These reports suggest that biological functions of protein polysulfidation differ for each protein and that this modification may be related to diverse cellular processes under physiological and pathological conditions.

## 5. Conclusions

Current detection methods of reactive persulfide species enable researchers to readily identify and investigate these molecular species both in vitro and in vivo. In this review article, we described the intracellular abundance and significance of the LMW reactive persulfide species CysSSH and GSSH and the HMW reactive species polysulfidated proteins. As our earlier work demonstrated, CBS and CSE may contribute to endogenous generation of reactive persulfide species. However, alternative pathways or biosynthetic systems of reactive persulfide species may still exist, distinct from CBS and CSE in cells, because CBS/CSE knocked down by small interfering RNA did not completely halt intracellular CysSSH production, and marked CysSSH generation was detected even in cardiac myocytes with very low CBS and CSE expression [13].

Reactive persulfide species possess markedly high antioxidant and nucleophilic properties. These reactive persulfide species are critically involved not only in the detoxification of environmental electrophiles but also in the regulation of redox signaling by means of 8-nitro-cGMP metabolism. However, details of the decomposition mechanism of protein polysulfidation are not fully known. Dóka et al. recently proposed a mechanism of protein polysulfide regulation by thioredoxin (Trx)/Trx reductase and/or glutaredoxin/glutathione reductase/GSH machinery [20]. They reported that the Trx and GSH systems may independently catalyze reductions of protein polysulfides, thereby fine-tuning persulfide signaling pathways [20]. Experimental evidence of protein *S*-guanylation by 8-nitro-cGMP and its redox signaling regulation by reactive persulfide species may thus warrant additional innovative research that may stimulate a new era of reactive thiol-mediated redox biology and the related chemical sciences of physiology, pathophysiology, and pharmaceutical chemistry, which may promote the development of novel therapeutics for oxidative stress–related diseases.
